# Quantitative Analysis of the Acceptance and Learning Success Instead of Flipped Classroom Teaching in a Caries Diagnosis Course for Undergraduate Students

**DOI:** 10.1155/2022/7749638

**Published:** 2022-11-16

**Authors:** Mozhgan Bizhang, Stefan Zimmer, Jan P. Ehlers

**Affiliations:** ^1^Faculty of Health, Department of Dental Medicine, Department of Operative and Preventive Dentistry, Witten/Herdecke University, Alfred-Herrhausen-Str. 50, Witten 58448, Germany; ^2^Didactics and Education Research in the Health Sector, Faculty of Health, Witten/Herdecke University, Alfred-Herrhausen-Str. 50, Witten 58448, Germany

## Abstract

This pilot study aimed to investigate the effectiveness of a flipped classroom for undergraduate students in dentistry. The main objective was to compare the knowledge level of students before and after lectures and practice. All second-year dental students (*n* = 44) at Witten/Herdecke University participated in this pilot study. They took four knowledge assessments, i.e., T0: the baseline, T2a: after the online lecture (two weeks after T0), T2b: immediately after the face-to-face session, and T3: after the practical session (three weeks after T2). The students' satisfaction and self-assessment of their abilities were determined immediately after the practical session in an anonymous online questionnaire using LimeSurvey. To assess the level of knowledge, we used the Friedman and Wilcoxon-signed-rank tests with the Bonferroni correction to analyze the correct answer by comparing the results from different sessions. The students' satisfaction and self-assessment of their abilities were determined descriptively, presenting the mean and standard deviation. A significance level of *p* ≤ 0.05 was applied. Data from thirty-nine students regarding the level of knowledge were analyzed. There were statistically significant differences in the level of knowledge of the students at different times (*p* = 0.001). A total of 19.5% of students reported a problem with the flipped classroom method, and 80.5% reported no problem with this educational method. Ninety-four percent of students would like further flipped classrooms in dental education. Within the limitations of this pilot study, the results suggest that dental students benefit from the flipped classroom method and that this mode of education can be effective in introducing caries diagnosis education for undergraduate students.

## 1. Introduction

Traditionally, the face-to-face lecture-based method has been widely used to teach undergraduate dental students. The new regulation of licensing for dentists in Germany has been in effect since October 2021 [1]. In this regulation, more self-study for students was required at the expense of in-person lectures. The overall goal is to reduce the number of in-person lectures in dentistry by approximately 50%.

In addition, new modern teaching methods with more student activity in the classroom should be implemented instead of frontal teaching. The flipped classroom method is characterized by preclass and in-class activities. The advantage of the flipped classroom is that it is an active learning environment in which students are encouraged to participate in the learning process during class sessions and spend less time during in-class sessions [2]. The aim of the flipped classroom is to shift the classic explanatory phase of face-to-face lecturing to self-study [3], thus creating more time for interactive learning with students in a subsequent session. In the original concept of the flipped classroom, teachers deliver the lecture via audio or video [4]. However, digital material featuring actors may also be offered. The concept is developed on certain aspects of educational psychology that assume there are higher-quality and lower-quality cognitive processes [5]. Therefore, this method encourages students to actively participate in the learning process and critical thinking. Furthermore, teachers have the chance to transfer theoretical knowledge to students in the classroom [6, 7]. Hew and Lo suggested that the flipped classroom shows a significant improvement in student learning compared to traditional teaching methods [8, 9].

On the other hand, a review reported that some students were dissatisfied with this approach to learning with an increased effort involved in preclass active learning and preparation for the lecture (6). The literature shows controversial results for comparisons between flipped classroom and traditional lectures. One study reported that students in a traditional classroom performed significantly better than their flipped classroom counterparts did. The results showed that students generally increased in their perceived easiness rating from presemesterto postsemester, but students attending traditional lectures experienced the greatest increase. [10]. Flipped classrooms have also been reported in different fields of dentistry. One study showed that 85% of students would have liked to have had more flipped lectures in the predoctoral dental curriculum. These students had more fun and were able to be more interactive in their second year of dental anatomy than students from traditional lectures. However, one of the noted disadvantages of this teaching method is the requirement of an Internet connection [11]. Two studies on flipped classrooms in the field of pediatric dentistry showed that students were satisfied with this method [12, 13], providing the notable advantages of availability and access to online content and course materials, clickers to test knowledge in preparation for an exam, and in-class group discussions with the help of students questionnaires [12]. The results of a study with a flipped classroom on the physiology of the autonomic nervous system indicated that students spent more time learning, understood the content better, performed better in assessments, and preferred this method to a traditional lecture [14]. Another study on online lectures in dermatology showed that these were highly welcomed by students and may be a good means to improve the education of students in dermatology. At the same time, however, the study found no difference between traditional lectures and online lectures [15].

A literature research study on the use of flipped classrooms in the field of caries diagnosis revealed no studies in this area to date. Second-year dental undergraduate students at Witten/Herdecke University (UWH) are taught caries diagnosis in a preclinical course to be able to put this theoretical knowledge to use in clinical courses. To date, this knowledge has been transmitted to students mainly via face-to-face lectures and practical courses. Fortunately, we know from previously conducted surveys that UWH dentistry students are very well equipped in terms of digital devices and Internet connections [16]. We postulate that students taught in the flipped classroom will have a better understanding of caries diagnosis and therefore will find it easier to apply to daily dental practice.

The aim of this pilot study was to evaluate the undergraduates' experience using the flipped classroom method, including their knowledge and satisfaction level with this form of dental teaching.

### 1.1. Outcome Measures

The primary endpoint was to determine the level of knowledge over different teaching sessions. The students' satisfaction and self-assessment of their abilities were also defined as secondary endpoints.

## 2. Materials and Methods

### 2.1. Ethical Clearance

The Ethics Committee of Witten/Herdecke University granted ethical approval for the pilot study (number: S-261/2021). All participants provided written informed consent.

### 2.2. Participants

Lectures in preventive dentistry at Witten/Herdecke University are held over five semesters (1st–5th semester). For this pilot study, a lecture in preventive dentistry (caries diagnosis) was converted to the flipped classroom method. All second-year dental students in the third semester (winter semester) participated in this pilot study (*n* = 44). These students were selected for the pilot study as they had prior experience with face-to-face teaching for two semesters (cf. historical comparison), thus allowing them to assess the new method more objectively.

### 2.3. Study Design and Class Activity (Face-to-Face Activity, T0)

Two weeks prior to the commencement of the online lecture, students were informed about the principles of the flipped classroom method, the intended time period of the pilot study, and the pilot study schedule at an information event. Furthermore, the knowledge levels of students regarding caries diagnosis were assessed at this meeting using twenty questions: ten Type A MCQs (single select multiple choice questions) and ten Type B SAQs (short answer questions). Students were additionally motivated to prepare a PowerPoint presentation with an audio recording for the next face-to-face phase.

### 2.4. Online Education (T2a)

The subject of “caries diagnosis” consists of a theoretical and a practical part, both of which were carried out by a lecturer (MB). The 90-minute PowerPoint lecture on “Caries Diagnosis,” with an audio recording, included three sessions of 30 minutes each. The lecture included a detailed description of caries and its localization, different methods for caries diagnosis, and the sensitivity and specificity of methods and their indications. To retain the students' attention, a small quiz was incorporated every 30 minute. The lecture was uploaded on the learning platform “Moodle.” This platform is freely accessible to all students of Witten/Herdecke University. The students participating in the pilot study were given two weeks to prepare for the next face-to-face teaching session [17].

### 2.5. Class Activity (Face-to-Face Activity, T2b)

**Figure 1:**
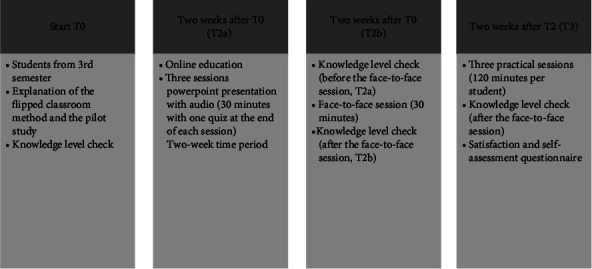
Flipped classroom schedule.

Two weeks following the upload of the lecture on Moodle, the face-to-face class activity started with a quiz to encourage a discussion and thus the active participation of the students in the session. This time was intended for students to deepen their core knowledge for questions and answers and for discussion of the topic with the help of clinical examples. This session had a duration of 60 minutes [18, 19].  Figure 1 shows the study schedule (Figure 1).

### 2.6. Practical Activity (T3)

The practical session took place two weeks following the face-to-face session in three appointments. Forty-four students were divided into groups, with three participants in each group. They implemented their knowledge of caries diagnosis by carrying out a dental examination and documenting the dental caries status [20]. This practical clinical session had a duration of 120 minutes and took place in the treatment rooms of the dental hospital at Witten/Herdecke University. The lecturer (MB) checked the documentation of the caries status.

### 2.7. Assessing the Knowledge Level

The first knowledge level test (after the information session, T0) was carried out on paper because students did not have their access data for registration on Moodle. The second (before the face-to-face session, T2a), third (after the face-to-face session, T2b), and fourth test (after the practical session, T3) took place digitally. Thereafter, students answered the same twenty knowledge questions at every session. The increase in the level of knowledge was recorded using twenty dental questions in which students had to diagnose caries. The knowledge level test consisted of ten theoretical Type A MCQs and ten Type B SAQs involving clinical situations, either clinical photographs or radiographs. The tests were created by the lecturer (MB), who has 20 years of teaching experience in this subject. Each correct question was weighted with 1 point, and the maximum score was 20.

### 2.8. Questionnaire of Satisfaction and Self-Assessment

The students' satisfaction and self-assessment of their abilities were determined immediately after the practical session with the help of LimeSurvey [20]. For this purpose, questions were used partly from the validated DREEM questionnaire [21, 22] and partly from validated GMA questionnaires [23]. LimeSurvey enabled the questionnaires to be filled out immediately, anonymously, and easily using a smartphone. Data were evaluated at the end of the pilot study period. Problems and possible solutions were documented for the successful establishment of the flipped classroom method over the long term in dental courses. Before starting the survey, students completed an informed consent form to acknowledge the information they had received regarding the survey and their voluntary participation in the survey. The survey consisted of demographic questions (sex and age), thirty items scored on a 5-point Likert scale (strongly agree = 1 to strongly disagree = 5), and two open-ended questions about the students' perception of the benefits and disadvantages of the flipped classroom. The questionnaire focused on the structure of the course, materials supplied, face-to-face lectures, and students' interest in and satisfaction with the course and the flipped classroom method.

### 2.9. Data Analysis

Data were statistically analyzed with SPSS® version 26.0. The Kolmogorov–Smirnov and Shapiro–Wilk tests were used to assess variables for normal distribution. As the results were significant, nonparametric tests were applied for evaluations. The results of different times (T0, T2a, T2b, and T3) were compared to assess the effectiveness of the flipped classroom method. The level of knowledge (analysis of correct answers) at different times was compared using both the Friedman and Wilcoxon-signed-rank tests. The Friedman test was used as a global test between all four examinations (T0, T2a, T2b, and T3), and the Wilcoxon-signed-rank test with the Bonferroni adjustment was used to evaluate differences between two examinations. A significance level of *p* ≤ 0.05 was applied. The students' satisfaction and self-assessment of their abilities were assessed in a postcourse survey questionnaire. These data were analyzed descriptively and shown as a mean and standard deviation.

## 3. Results

Thirty-nine of the forty-four students fully participated in this pilot study. The reason for the dropout of four students was their absence in the face-to-face activity, and one student was absent in the practical session. Overall, 56.4% of students were females, and the mean age was 22.69 years (SD ± 2.50, range 18.92–30.30 years).

### 3.1. Students' Knowledge Level for Different Time Intervals

**Figure 2:**
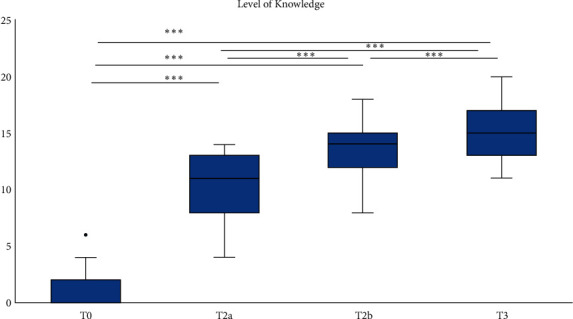
Boxplot of the knowledge level of students for different time intervals. T0 = before online education; T2a = before the face-to-face session; T2b = after the face-to-face session; T3 = after the practical session. ^*∗∗∗*^*p* < 0.001: Friedman and Wilcoxon-signed-rank tests indicate statistically significant differences.

Data were not normally distributed (Kolmogorov–Smirnov and Shapiro–Wilk tests). There were statistically significant differences in the level of knowledge of students in the four examinations (Friedman and Wilcoxon-signed-rank tests, *p* = 0.001). The Friedman test was used as a global test between all four examinations (T0, T2a, T2b, and T3) and was significant (*p* = 0.001). The Wilcoxon-signed-rank test with the Bonferroni adjustment was used to evaluate multiple pairwise comparisons between four sessions. The differences were *p*found to be significant (*p* = 0.001).  Table 1 shows the descriptive statistics of the level of education for four examination times. The median (25–75 percentiles for the level of knowledge) was 0.00 (0.00–2.00) for the first time (T0, two weeks before the face-to-face lecture), 11.00 (8.00–13.00) for the second time (T2a, immediately before the face-to-face lecture), 14.00 (12.00–15.00) for the third time (T2b, after the face-to-face lecture), and 15.00 (13.00–17.00) for the final time (T3, after the practical session). The median (25–75 percentiles) was 10.00 (7.00–12.00) for the interval between T0 and T2a, and it was 3.00 (1.00–5.00) between T2a and T2b and 2.00 (1.00–3.00) between T2b and T3. Statistically significant differences were found pairwise between the four sessions. The Friedman test as a global test and the pairwise Wilcoxon-signed-rank test with the Bonferroni adjustment were used (*p* = 0.001).  Figure 2 shows the knowledge level of students at four different times: T0, T2a, T2b, and T3. Statistically significant differences were found pairwise between the four sessions (*p* < 0.001).

The passing level was set at 12 points out of 20. None of the students passed the dental quiz at T0, but 19 (48.7%) students at T2a, 31 (79.5%) students at T2b, and 38 (97.4%) students at T3 passed the examination.

### 3.2. Students' Satisfaction and Self-Assessment of Their Abilities

LimeSurvey was performed anonymously. Forty-two students completed the online survey after the practical session. The results are presented in Table 2. Most students reported that they liked the flexibility of being able to study with the online session. They found it easy to study the teaching material at a time suitable to them and to repeat or revise the material several times. They were able to implement it well, as the practical session based on lecture topics helped them enormously understand and memorize the lecture. They found this method of teaching more versatile than face-to-face teaching. A total of 80.5% of students reported no problems with this education method, while 19.5% reported problems with the flipped classroom method and preferred the frontal lecture. Ninety-four percent would like to have further flipped classrooms in dental education, whereas 6% preferred traditional lectures. Students had the following suggestions for improvement: they expressed the need to be able to adjust the speed of the audio playback and would like to obtain better notifications regarding the dates for tests. These results suggested that the success of this teaching method heavily depended on the motivation of students. Two students requested the need for more than two weeks for online education; on the other hand, one student preferred less than two weeks. However, the mean of the answer to technical problems during the course was 4.02 (disagree).

### 3.3. Quiz Time

The first test required approximately 5 minutes (paper version). The knowledge of students was very limited at the baseline (T0); therefore, they were able to answer very quickly. The times required for additional tests are described in Table 3. The students spent less time at T3 than at T2a or T2b.

## 4. Discussion

This pilot study evaluated the effectiveness of a flipped classroom in a caries diagnosis course for undergraduate second-year dental students. This pilot study showed an increase in the level of knowledge of students, and most students preferred the flipped classroom to the traditional teaching method, which they had experienced in preceding semesters.

Forty-four students were recruited for the pilot study; however, data analysis was carried out for 39 students who participated in all sessions. This pilot study was conducted during the SARS-COV-2 pandemic in Germany. In accordance with the university policy, all students with signs or symptoms of a cold, sore throat, or flu were instructed not to participate in face-to-face sessions. The total number of students in the present semester was 44 and thus limited the planning for two groups. However, since the participating students had experience with traditional teaching in previous semesters, they were well able to assess the benefits and disadvantages of the flipped classroom method. Furthermore, in accordance with the SARS-COV-2 regulations, students from early semesters had attended solely online lectures and had different questions to evaluate the education level. Therefore, they could not be taken into consideration for the control group.

Our results showed that students performed significantly better in Quiz T2a than in Quiz T0. Some of the students did not study the teaching material through self-study alone; however, a large majority of the group followed online education. According to our results, students were more active and cooperative with the flipped classroom method. Our results were found to be consistent with those of other studies [24–26].

As the same questions were applied to all sessions, one could speculate that this had an influence on the improvement of the students' level of knowledge. The interval of two or three weeks of the knowledge level test was maintained to counteract this gap. On the other hand, the lecturer (MB) did not discuss correct answers with the groups. Hence, the influence might not be considered to be great, as shown in other studies [27], nevertheless, even with the influence being low. Concurrently, it must be stated that students were unaware of this part of the pilot study schedule and therefore felt no necessity to memorize quiz questions. Moreover, questions, answers, and results were at no point shared with students, thus preventing any bias. Similarly, this concept was also used in another study [14, 24].

The knowledge level test consisted of ten single choice theoretical questions and ten clinical situations, presented as clinical photographs or radiographs. Each correct question was weighted with 1 point, with the maximum score being 20. The same quiz questions were applied to test the knowledge level after each session. As a means to assess knowledge development over the pilot study period, it was essential that the same questions be utilized in the tests [14]. The greatest increase in the knowledge level of students was found to be after the online lecture, and a continuous increase in the level of knowledge after each session was achieved. The same observation was found by Hew and Lo [8] in a meta-analysis, showing a significant overall effect in favor of the flipped classroom when compared to face-to-face teaching used in the education of health professionals [8, 28]. In the present pilot study, the students were found to have deepened their knowledge of caries diagnosis after the practical session, which took place three weeks after the face-to-face session, thus attaining a long-term increase in knowledge. One study on students who took part in a combination of simulation and flipped classroom confirmed that knowledge was improved in the short term, but they also retained this knowledge over the long term [29].

Data analysis of LimeSurvey showed that most of the students were satisfied with online education, and it was possible for them to regulate their learning speed and individual needs by pausing, rewinding, or replaying online education in full or in part. In addition, students found that the three sections of online education, each with a length of 30 minutes, were quite suitable. A PowerPoint presentation accompanied by an audio narration of the presentation was uploaded to the online learning platform Moodle. Some students, however, reported a preference for YouTube videos as a means for online education. This suggestion should be explored in further studies. A review article concerning flipped classrooms in dental education showed that the majority of students had a positive opinion toward this form of teaching [30]. Furthermore, the flipped classroom has been introduced in different fields of dentistry. Students considered it a more fun interactive and collaborative technique than the traditional class [13, 14, 31].

Despite positive results, this pilot study has several limitations. Due to the limited number of students in the semester (*n* = 44), it was not possible to establish a control group for this pilot study. Another drawback was the lack of an online platform for students to support discussions among themselves and between them and the lecturer during session intervals. Furthermore, only one question was added at the end of each session in the PowerPoint presentation for self-assessment. Finally, another limitation was that the first quiz used to test the knowledge level was carried out on paper; therefore, these data could not be analyzed.

The concept of the flipped classroom was well accepted by the majority of students. The desire expressed by the students in this pilot study to be able to attend more lectures with this method in dentistry has been confirmed by other studies in different educational areas [8, 17, 19]. This study was, however, an investigation comprising 44 students in the field of preventive dentistry and cannot necessarily be generalized to other specialties in dentistry. Future trials should focus on the efficacy of using flipped classrooms in other areas of dentistry, such as root canal treatment and pediatric or prosthetic dentistry.

## 5. Conclusions

Within the limitations of this study, the results showed that implementing the flipped classroom in a dental caries diagnosis course improved student knowledge and satisfaction level with this form of dental teaching. It can be concluded that the flipped classroom method provided a positive experience for dental undergraduate students. Future investigations in additional dental courses including a bigger sample with a control group should be carried out to confirm these results.

## Figures and Tables

**Table 1:** Descriptive statistics of the knowledge level between different time intervals (mean and standard deviation (SD)).

	N	Mean	SD	Min.	Max.	Percentiles		
						25th	50th (median)	75th
T0_knowledge	39	1.00	1.50	.00	6.00	.00	.00	2.00
T2a_knowledge	39	10.51	2.76	4.00	14.00	8.00	11.00	13.00
T2b_knowledge	39	13.44	2.52	8.00	18.00	12.00	14.00	15.00
T3_knowledge	39	15.10	2.28	11.00	20.00	13.00	15.00	17.00

T0 = before online education; T2a = before the face-to-face session; T2b = after the face-to-face session; T3 = after the practical session.

**Table 2:** Mean (standard deviation) of the final questionnaire about the students' responses to the use of the flipped classroom (strongly agree = 1–5 = strongly disagree).

Final questionnaire	Mean	SD
The teaching time was put to good use	1.81	0.96
The teaching was clearly structured	1.67	0.85
I was clear about the learning objectives of the course	1.72	0.97
I was able to use the teaching platform Moodle	1.89	1.01
The teaching materials were made available in good time before the face-to-face lecture	1.49	0.93
The teaching time was enough to study the online lecture	1.72	0.97
The length of the presentation was sufficient	1.57	0.83
There were often technical problems during the course	4.02	1.13
My skills in using digital tools were sufficient	1.28	0.50
It was easy for me to structure and organize myself independently	1.81	0.95
I felt well looked after during the online activity	2.00	0.83
Teaching encouraged me to be an active learner	1.55	0.83
The teacher was good at providing feedback to students	1.51	0.80
The teacher was knowledgeable	1.19	0.50
The teacher was well prepared for the topic in the course in a clear and understandable way	1.77	0.87
I found the difficulty of the self-study task appropriate	1.37	0.49
I was encouraged to take an active part in the course	2.07	0.89
Teaching encouraged active learning for me	1.96	0.95
The atmosphere was relaxed during the lecture	1.56	0.62
I felt I was able to ask questions I wanted to ask	1.60	0.72
I felt comfortable in class socially	1.42	0.62
Enjoyment outweighed the stress of the course	1.78	0.85
The atmosphere motivated me as a learner	1.96	0.82
I was able to concentrate well	1.76	0.83
The atmosphere during the practical session was pleasant	1.41	0.66
I rate the teaching format “flipped classroom” with the following overall grade	1.80	0.63
The “flipped classroom” teaching format contributed to my learning success	1.86	0.85

**Table 3:** Time required for the knowledge level tests at different sessions.

	T2a: before the face-to-face session	T2b: after the face-to-face session	T3: after thepractical session
Minimum	4.29	8.00	5.45
Maximum	20.50	18.00	22.41
Mean	14.37	13.44	12.02
SD	3.28	2.52	3.73

## Data Availability

The research data used to support this study are included within the article.

## Supplementary Materials

Supplementary Materials The questionnaire flipped classroom showed the perception of the communicative, instrumental, and pedagogical potential of the flipped classroom.
