# Supplementary material for: Quantitative Analysis of the Acceptance and Learning Success Instead of Flipped Classroom Teaching in a Caries Diagnosis Course for Undergraduate Students
Source: Int J Dent. 2022 Nov 16;2022:7749638. doi: 10.1155/2022/7749638 (PMC9683971; doi:10.1155/2022/7749638)
Supplement: Supplementary Materials — The questionnaire flipped classroom showed the perception of the communicative, instrumental, and pedagogical potential of the flipped classroom. [file 7749638.f1.pdf]

“Flipped Classroom” Caries Diagnosis

Satisfaction Questionnaire

Sex: ☐ female ☐ male ☐ diverse
Age: ☐ ≤ 20 ☐21-25 ☐26-30
☐31-35 ☐ > 35 ☐ no answer

Communication (from digital evaluation GMA)

- **The teaching time was put to good use**
  strongly agree ☐ ☐ ☐ ☐ ☐ strongly disagree ☐ no answer
- **The teaching was clearly structured**
  strongly agree ☐ ☐ ☐ ☐ ☐ strongly disagree ☐ no answer
- **I was clear about the learning objectives of the course**
  strongly agree ☐ ☐ ☐ ☐ ☐ strongly disagree ☐ no answer
- **I was able to use the teaching platform Moodle**
  strongly agree ☐ ☐ ☐ ☐ ☐ strongly disagree ☐ no answer
- **The teaching materials were made available in good time before the face-to-face lecture**
  strongly agree ☐ ☐ ☐ ☐ ☐ strongly disagree ☐ no answer
- **The length of the presentation was sufficient**
  strongly agree ☐ ☐ ☐ ☐ ☐ strongly disagree ☐ no answer
- **The teaching time was enough to study the online lecture**
  strongly agree ☐ ☐ ☐ ☐ ☐ strongly disagree ☐ no answer

Technology (from digital evaluation GMA)

- **There were often technical problems during the course**
  strongly agree ☐ ☐ ☐ ☐ ☐ strongly disagree ☐ no answer
- **My skills in using the digital tools were sufficient**
  Strongly agree ☐ ☐ ☐ ☐ ☐ strongly disagree ☐ no answer
- **It was easy for me to structure and organize myself independently**
  strongly agree ☐ ☐ ☐ ☐ ☐ strongly disagree ☐ no answer

Support (from digital evaluation GMA)

- **I felt well looked after during the online activity**
  strongly agree ☐ ☐ ☐ ☐ ☐ strongly disagree ☐ no answer
- **The teaching encouraged me to be an active learner**
  strongly agree ☐ ☐ ☐ ☐ ☐ strongly disagree ☐ no answer
- **The teacher was good at providing feedback to students**
  strongly agree ☐ ☐ ☐ ☐ ☐ strongly disagree ☐ no answer
- **The teacher was knowledgeable**
  strongly agree ☐ ☐ ☐ ☐ ☐ strongly disagree ☐ no answer
- **The teacher was well prepared for the topic in the course in a clear and understandable way**
  strongly agree ☐ ☐ ☐ ☐ ☐ strongly disagree ☐ no answer
- **I found the difficulty of the self-study task appropriate**
  strongly agree ☐ ☐ ☐ ☐ ☐ strongly disagree ☐ no answer

Teaching (from BLOOM)

- **I was encouraged to take an active part in the course**
  strongly agree ☐ ☐ ☐ ☐ ☐ strongly disagree ☐ no answer
- **The teaching encouraged active learning for me** strongly agree ☐ ☐ ☐ ☐ ☐ strongly disagree ☐ no answer

Atmosphere (from BLOOM)

- **The atmosphere was relaxed during the lecture**
  strongly agree ☐ ☐ ☐ ☐ ☐ strongly disagree ☐ no answer
- **I felt able to ask the questions I wanted to ask**
  strongly agree ☐ ☐ ☐ ☐ ☐ strongly disagree ☐ no answer
- **I felt comfortable in class socially**
  strongly agree ☐ ☐ ☐ ☐ ☐ strongly disagree ☐ no answer
- **The enjoyment outweighed the stress of the course**
  strongly agree ☐ ☐ ☐ ☐ ☐ strongly disagree ☐ no answer
- **The atmosphere motivated me as a learner**
  strongly agree ☐ ☐ ☐ ☐ ☐ strongly disagree ☐ no answer
- **I was able to concentrate well**
  strongly agree ☐ ☐ ☐ ☐ ☐ strongly disagree ☐ no answer
- **The atmosphere during the practical session was pleasant**
  strongly agree ☐ ☐ ☐ ☐ ☐ strongly disagree ☐ no answer

Overall Rating (from digital evaluation GMA)

- **I rate the "flipped classroom" teaching format with the following overall grade**
  1 ☐2 ☐ 3☐ 4☐ 5☐ ☐ no answer
- **The "flipped classroom" teaching format contributed to my learning success**
  strongly agree ☐ ☐ ☐ ☐ ☐ strongly disagree ☐ no answer
- **I have the following points of praise, criticism and improvement:**
  ____
  ____
  ____
  ____
  ____

Thank you very much for participating in my project and for taking the time to fill out the questionnaire!
